# Waiting for a better possibility: delay of gratification in corvids and its relationship to other cognitive capacities

**DOI:** 10.1098/rstb.2021.0348

**Published:** 2022-12-19

**Authors:** Alexandra K. Schnell, Markus Boeckle, Nicola S. Clayton

**Affiliations:** ^1^ Department of Psychology, University of Cambridge, Cambridge CB2 3EB, UK; ^2^ Scientific Working Group, Karl Landsteiner University of Health Sciences, D.O.T. - Die offene Tür (The open door), Krems, Austria

**Keywords:** inter-temporal choice task, delay maintenance, self-control, cognitive evolution, convergent evolution, general intelligence

## Abstract

Self-control, the ability to resist temptation and wait for better but delayed possibilities, is an important cognitive skill that underpins decision-making and planning. The capacity to exert self-control has been linked to intelligence in humans, chimpanzees and most recently cuttlefish. Here, we presented 10 Eurasian jays, *Garrulus glandarius*, with a delayed maintenance task, which measured the ability to choose a preferred outcome as well as the ability to sustain the delay prior to that outcome. Jays were able to wait for better possibilities, but maximum wait times varied across the subjects. We also presented them with five cognitive tasks that assessed spatial memory, spatial relationships and learning capacity. These tasks are commonly used as measures of general intelligence within an ecological context. Individual performance was correlated across the cognitive tasks, which suggests that there was a general intelligence factor underlying their performance. Performance in these tasks was correlated significantly with the jays' capacity to wait for better possibilities. This study demonstrates that self-control and intelligence are correlated in jays. The fact that this correlation exists in diverse species suggests that self-control is a fundamental feature of cognition. Our results are discussed in the context of convergent evolution.

This article is part of the theme issue ‘Thinking about possibilities: mechanisms, ontogeny, functions and phylogeny’.

## 1. Introduction

Individuals are often faced with decisions that influence what rewards or options become available in the future. The ability to resist immediate rewards and wait for better possibilities is termed self-control, an important cognitive skill that underpins decision-making, agency and future planning [1–7]. Self-control is cognitively challenging as individuals must not only resist temptation in the present moment but also override the tendency to temporally discount or devalue future rewards [8]. This ability is typically measured using delay of gratification tasks such as the classic marshmallow test [9], where subjects are required to choose between two options: a less preferred reward, which is available immediately, or a better reward, which is only available after a delay. Performance in such tasks can be highly variable across individuals. In humans, there is a clear relationship between the ability to delay gratification and general intelligence [9–13]. For example, children that can resist temptation for longer also attain higher scores in a range of academic tasks, which are proxy measures of general intelligence in humans [14–17].

The association between self-control and intelligence does not appear to be limited to humans. Recent research on non-human animals has demonstrated a similar relationship. For instance, one study on chimpanzees showed that individuals which exert greater self-control show better performance across 13 different cognitive tasks [18]. Another study demonstrated that cuttlefish which delay gratification for longer show better learning performance in a reversal-learning task [19]. Given the considerable evolutionary divergence between chimpanzees and cuttlefish, evidence of such an association suggests that self-control and its relationship to other cognitive abilities evolved independently. It also raises the question as to whether this relationship exists in other animal groups.

The ability to delay gratification not only varies across individuals but also across different animal species. Common marmosets, *Callithrix jacchus,* and cottontop tamarins, *Saguinus oedipus,* [20] as well as domestic fowl, *Gallus gallus domestricus*, [21] and pigeons, *Columba livia,* [22] typically favour the immediate option even when the delayed option results in a reward of higher value. By contrast, great apes [23–25], cuttlefish [19] and large-brained birds such as corvids (crow family) [26–28] and psittacines (parrot order) [29] are among the animal groups that demonstrate advanced self-control, commonly ignoring immediate options and waiting for up to several minutes for better but delayed rewards [30]. Specific ecological or social pressures are often highlighted as the drivers of advanced self-control in these disparate groups [30].

In corvids, caching behaviour, i.e. hiding food for later consumption, offers an illustrative example of how ecological pressures such as the need to inhibit immediate gratification to plan for future meals may have driven the evolution of self-control [31,32]. Caching behaviour also imposes social pressures that might have reinforced the evolution of self-control. For instance, some species of corvids such as crows, ravens and jays, are vulnerable to cache theft. These species use strategies that require self-control to minimize the chance of conspecifics stealing their caches, such as waiting for the optimal moment to make a cache*,* i.e. when a competitor is out of sight or out of earshot [33–38].

Corvids are also known for their cognitive abilities and appear to rival the great apes in many psychological domains [39]. This makes them an ideal candidate for investigating the prevalence of the relationship between self-control and other cognitive abilities. Here, we investigate this relationship in Eurasian jays, *Garrulus glandarius*, a food-caching corvid that is renowned for overcoming both ecological and social challenges using cognitive solutions that are likely to require self-control. We present 10 Eurasian jays with two experiments. In experiment 1, we use five cognitive tasks to assess areas of physical cognition including spatial memory, understanding of spatial relationships such as object permanence, and learning capacity including generalization, discrimination and reversal learning. These tasks are commonly used as measures of general intelligence within the context of ecological cognitive skills [18] (electronic supplementary material). In experiment 2, we use an inter-temporal delay maintenance task, which measures an individual's ability to choose a preferred outcome (i.e. choose between less preferred and preferred food) as well as their ability to sustain the delay prior to the outcome (i.e. wait for the preferred food despite the time delay) [40,41]. We compare performance across both experiments to assess whether a relationship between self-control and general intelligence is present in Eurasian jays.

## 2. Methods

### (a) Subjects

Ten sub-adult Eurasian jays (3-years old) from a long-term, stable social group of 16 birds were used in this study from March to June 2017. Birds were housed at the Sub-Department of Animal Behaviour, University of Cambridge in Madingley, Cambridgeshire, UK. Housing consisted of an outdoor aviary (20 × 10 × 3 m) with indoor compartments (2 × 1 × 2 m) at one end. Birds were able to enter the indoor compartments from the aviary via opaque trap-windows (0.5 × 0.5 m), whereby access was controlled by the experimenter (electronic supplementary material).

### (b) Experiment 1: physical cognition

All jays completed a battery of cognitive tests prior to the self-control experiment. This experiment consisted of five tasks including spatial memory, object permanence, generalization learning, discrimination learning and reversal learning. Trial counts were minimized in all tasks because the battery was designed to assess spontaneous performance rather than trained performances. Trials were administered over several days of testing, depending on subject availability and motivation. Food rewards were offered to subjects for all operationally defined correct responses for each task (electronic supplementary material).

### (c) Experiment 2: self-control

#### (i) Experimental apparatus

**Figure 1.**
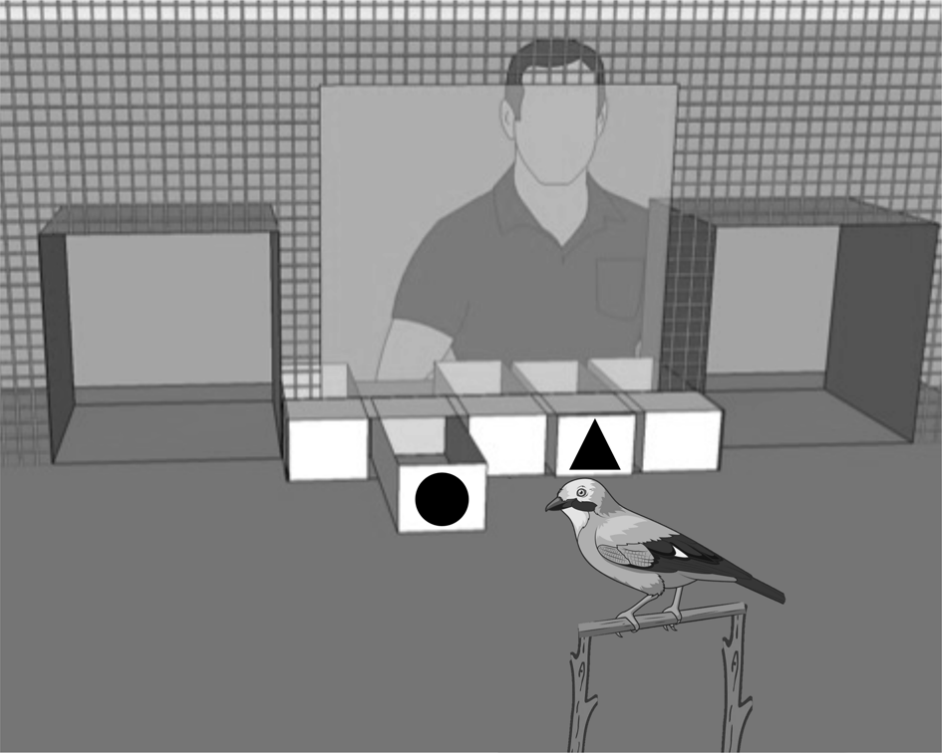
Schematic representation of the test conditions in the delay maintenance task: control condition and experimental condition. The different shaped visual symbols represent the time delays that were associated with each drawer. The immediate-release drawer (represented by a circle in the figure) was always baited with the less preferred food item whereas the delayed drawers (i.e. obtainable and unobtainable) were always baited with the preferred food item (image source: © Alexander Polusay stock.adobe.com).

We constructed a movable experimental apparatus consisting of five white plastic drawers 320 × 110 × 80 cm (*w* × *l* × *h*; dimensions of each drawer) with a transparent bench top so that the content of each drawer was visible. Each drawer was fitted with a detachable black plastic visual symbol that differed in shape so that jays learnt to associate a specific shaped symbol with different types of accessibility. The drawers were inserted horizontally below a testing window and directly in front of a testing perch (figure 1).

#### (ii) Preference tests and training

We conducted preference tests to determine individual food preferences in which we identified a preference rank for four food types. The most preferred, second preferred, and third preferred food items pertaining to each individual were subsequently used as their rewards in the self-control experiment (electronic supplementary material, table S1). We then trained subjects to learn to associate a specific shaped symbol with a different type of accessibility to the baited contents of the drawer. There were three types of accessibility conditions: (i) immediate and accessible food; (ii) delayed release with obtainable food following the delay; and (iii) delayed release with unobtainable food following the delay, whereby a second film of clear Perspex obstructed access to the food item once the drawer was pushed forward. We also trained subjects to learn that once they approached a drawer, the food item in the alternative drawer was removed immediately (electronic supplementary material).

#### (iii) Test phase: self-control

To test self-control, jays were presented with an inter-temporal delay maintenance task, which required them to choose between two food items of different quality. We included two food quality treatments: (i) most preferred versus second preferred, and (ii) most preferred versus third preferred. Jays also experienced two testing conditions including a control and an experimental condition. In the control condition, subjects were required to choose between an immediate food item and delayed but unobtainable food item. This condition allowed us to control for the possibility that the subjects were trained to learn to delay consumption of food across all conditions. The control condition also allowed us to assess whether the subjects found the less preferred food item desirable when they had visual access to their most preferred food but no physical access. In the experimental condition, subjects were required to choose between an immediate food item and delayed obtainable food item. This condition assessed whether the subjects were able to delay immediate gratification to obtain the preferred food. To determine the maximum amount of time that each subject was willing to wait for the preferred food item, we tested a range of delay times including 5, 10, 20, 30, 40, 60, 80, 160 and 320 s.

In both conditions, the immediate drawer was always baited with either the second or third preferred food item (i.e. piece of bread or cube of cheese) and the delayed drawer was always baited with the preferred food item (i.e. mealworm). After baiting, the immediate drawer was pushed open to expose the less preferred food, whereas the delay drawer remained closed. The delayed drawer would only open after the assigned delay if the subject had resisted the temptation of consuming the less preferred food in the immediate drawer. Subjects were able to discontinue waiting at any point and consume the less preferred food item, which remained visible and accessible throughout each trial. This set-up allowed us to measure the jays' ability to choose a preferred outcome (delay choice) as well as their ability to sustain the delay prior to that outcome (delay maintenance) [27]. Once subjects consumed the chosen food item (either the immediate or delayed), the food item in the alternative drawer was removed. For both conditions, we measured latency to consume any food item (either immediate or delayed). We expected jays to choose the immediate reward more often in the control condition and, by contrast, maintain the delay and wait for their preferred food item in the experimental condition. We, therefore, predicted that latency to consume any food item would be shorter in the control condition compared to the experimental condition. Since resisting temptation is probably influenced by the value of the immediate reward, we also predicted wait times to be significantly shorter in the preferred versus second preferred treatment compared to the preferred versus third preferred treatment.

### (d) Analysis

All data were high definition recorded and coded *in situ*, cross-referenced with the videos and then assessed for inter-rater reliability. Statistical analyses were completed using JASP (v.0.10.3, http://jasp-stats.org) and RStudio for MacOS (version 1.2.1335; R 4.1.1 binary for MacOS). To investigate performance in the physical cognition tasks, we calculated the percentage of correct responses for each task across trials (electronic supplementary material, table S2) and then analysed these measures with a principal components analysis (PCA). The PCA was conducted on five variables with an oblique rotation (oblimin) to determine whether performance in the physical cognition tasks was correlated and thus represented a broader dimension of intelligence. To determine whether the jays had preferences for different food items, we used binomial tests (against value: 0.5) (electronic supplementary material, table S1). To check parametric assumptions, we used Shapiro–Wilk normality tests (electronic supplementary material, table S3). To determine whether latency to consume a food item was influenced by delay (ordinal factor) or condition (nominal factor: control versus experimental), we used non-parametric permutation tests (aovperm function, permuco package). We also used the same test to determine whether latency to consume a food item was influenced by treatment (nominal factor: preferred_2^nd^ preferred versus preferred_3^rd^ preferred). To determine whether delay maintenance was correlated with delay duration, we used two-tailed Kendall's Tau B correlation coefficients. We used the same correlation coefficient, which is appropriate for small samples (less than 25) [42], to investigate whether physical cognition was correlated with self-control. The variables in the correlation included a ranked score for general performance in the physical cognition experiment and mean maximum wait time for each subject in the delay maintenance task. Given that we had a limited sample size of 10, we also examined these data to determine the strength of the evidence in support of the alternative or the null hypothesis. To do this, we calculated a Bayes factor (BF) using Bayesian information criteria [43], comparing the fit of the data under the null and the alternative hypothesis (BF_10_ = alternative/null).

## 3. Results

### (a) Experiment 1: physical cognition

A PCA was conducted on five variables (spatial memory, object permanence, generalization learning, discrimination learning and reversal learning) with an oblique rotation (oblimin). Bartlett's test of sphericity indicated that correlations between the variables were sufficiently large PCA ($\chi_{5}^{2}=13.06$, *p* < 0.05). One component had an eigenvalue over Kaiser's criterion of 1 and explained 93.4% of the variance in performance across the five tasks. The scree plot showed one point of inflexion, after component 1, suggesting that component 1 represents a general intelligence factor (table 1).

**Table 1.** Factor loadings for principal component analysis on the subjects' performance in five physical cognition tasks.

performance variable	RC1	uniqueness
spatial memory	0.976	0.047
object permanence	0.969	0.060
generalization learning	0.955	0.087
discrimination learning	0.958	0.082
reversal learning	0.972	0.055

Note: Applied rotation method is oblimin.

### (b) Experiment 2: self-control

#### (i) Food preference

Jays showed a preference order for the different food items. All subjects significantly preferred the mealworm when compared to all other food items on offer (binomial tests: mealworm versus bread *p* < 0.001; mealworm versus cheese *p* < 0.001; mealworm versus raisin *p* < 0.001). The second preferred food differed across individuals (electronic supplementary material, table S1): eight subjects preferred bread when compared to cheese (binomial test: *p* < 0.001) and two subjects significantly preferred cheese when compared to bread (binomial test: *p* < 0.001). For all subjects, raisin was their least preferred food item, with individuals significantly preferring both bread and cheese when compared to raisin (binomial tests: bread versus raisin *p* < 0.001; cheese versus raisin *p* < 0.001).

#### (ii) Latency to consume

**Figure 2.**
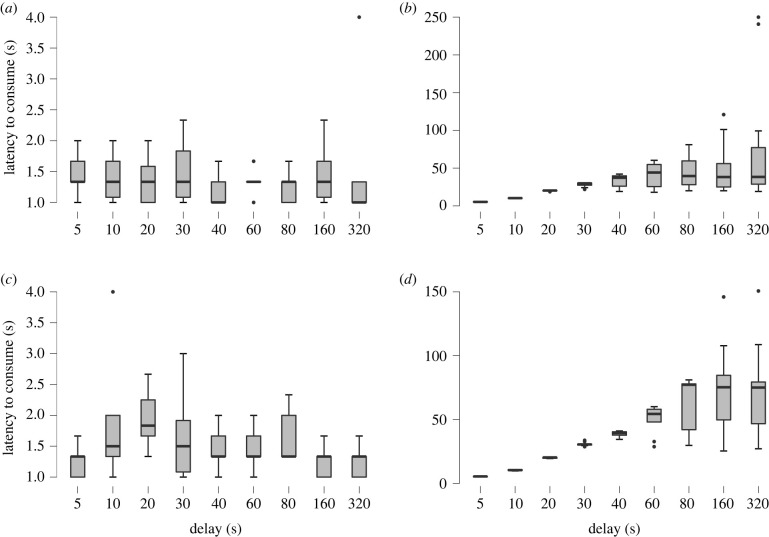
Latency to consume in Eurasian jays, *Garrulus glandarius* (*n* = 10). Mean latency to consume a food item (s) (mean ± s.e.) at different delay durations (5–320 s) in the (*a*) control (immediate, less preferred food versus delayed, unobtainable preferred food), and (*b*) experimental condition (immediate, less preferred food versus delayed, obtainable preferred food) when subjects were offered the most preferred versus second preferred food item. Mean latency to consume a food item (s) (mean ± s.e.) at different delay durations (5–320 s) in the (*c*) control (immediate, less preferred food versus delayed, unobtainable preferred food), and (*d*) experimental condition (immediate, less preferred food versus delayed, obtainable preferred food) when subjects were offered the most preferred versus third preferred food item.

When subjects were offered their most preferred food versus their second preferred food, latency to consume differed significantly between the conditions (*F* = 40.85; *p* < 0.001). Specifically, latencies to consume any food item were significantly longer in the experimental condition compared to the control condition (figure 2*a,b*). Similarly, when subjects were offered their most preferred food versus their third preferred food, latency to consume differed significantly between the conditions (*F* = 113.01; *p* < 0.001). Latencies to consume any food item were also significantly longer in the experimental condition compared to the control condition (figure 2*c*,*d*). The jays adjusted their latency to consume behaviour in response to the increased time delay, as demonstrated by the significant effect of delay duration (preferred versus second preferred: *F* = 6.38; *p* < 0.001; preferred versus third preferred: *F* = 22.96; *p* < 0.001). However, latency to consume was also dependent on foraging context, as demonstrated by the significant interaction between condition and delay duration (preferred versus second preferred: *F* = 6.49; *p* < 0.001; preferred versus third preferred: *F* = 23.38; *p* < 0.001). When we analysed the experimental trials, we found a significant effect of delay duration (*F* = 23.21; *p* <0.001) but no significant effect of treatment on latency to consume (*F* = 1.51; *p <* 0.239); the interaction between treatment and delay duration was also not statistically significant (*F* = 0.82; *p <* 0.598).

#### (iii) Delay maintenance

**Figure 3.**
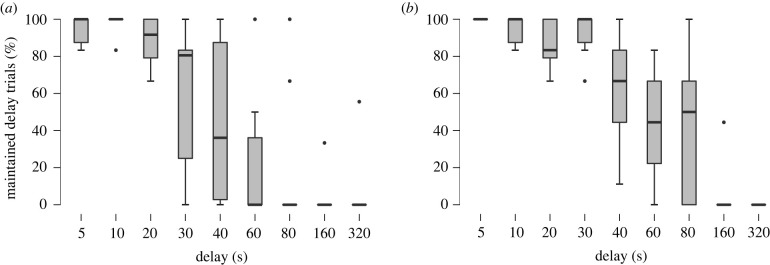
Delay maintenance in Eurasian jays, *Garrulus glandarius* (*n* = 10). Mean proportion of trials where subjects successfully maintained the delay (%) (mean ± s.e.), waiting for their preferred food at the different delay durations (5–320 s) when offered (*a*) most preferred versus second preferred food, and (*b*) most preferred versus third preferred food.

When subjects were offered their most preferred food versus their second preferred food in the experimental condition, delay maintenance was correlated significantly with delay duration (Kendall's Tau B = – 0.657; *p* < 0.001; figure 3*a*). A similar pattern was observed when subjects were offered their most preferred food versus their third preferred food (Kendall's Tau B = – 0.730; *p* < 0.001; figure 3*b*). Maximum wait time differed across subjects ranging from 20 to 320 s when subjects were offered their most preferred food versus their second preferred food and ranging from 40 to 160 s when subjects were offered their most preferred food versus their third preferred food (electronic supplementary material, table S4). Subsequent trials at increasing delay durations revealed that subjects often abandoned waiting at their maximum wait time (electronic supplementary material, delay maintenance data).

### (c) Self-control and physical cognition

**Figure 4.**
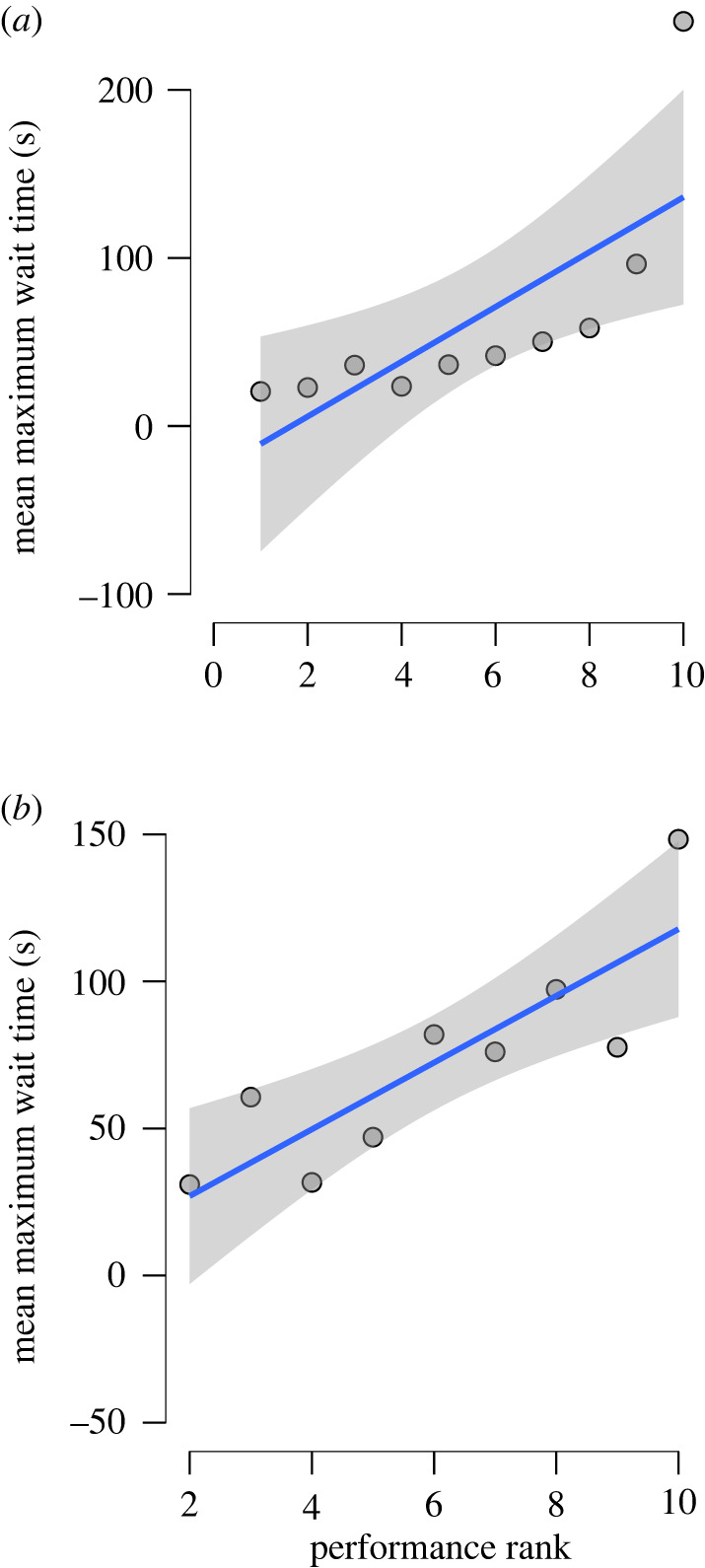
Delay maintenance and general intelligence in Eurasian jays, *Garrulus glandarius* (*n* = 10). Relationships between the performance rank across the five physical cognition tasks and mean maximum wait time (s) in the delay maintenance task when subjects were offered (*a*) most preferred versus second preferred food, and (*b*)* most preferred versus third preferred food. The relationships are indicated by two-tailed Kendall's Tau B correlation coefficient with 95% confidence intervals. Each data point represents a subject. * Notice that there are only nine data points in graph (*b*) because one subject, Jaylo, stopped consuming bread in the control trials, thus her data for this treatment was subsequently omitted. (Online version in colour.)

Jays that maintained delays for longer showed better general intelligence. Specifically, mean maximum wait time in the delay maintenance task was correlated significantly with the rank score for performance across all five physical cognition tasks (most preferred versus second preferred: Kendall's Tau B = 0.956, *p* < 0.001; most preferred versus third preferred: Kendall's Tau B = 0.722, *p* < 0.01; figure 4). The data were also analysed using a Bayesian correlation matrix, demonstrating that the strength of the evidence was strong in the most preferred versus second preferred treatment (BF_10_ = 209.59) and the strength of the evidence was moderate in the most preferred versus third preferred treatment (BF_10_ = 9.57). The BFs indicate that our data were 209.59 and 9.57 times more likely to be observed under the alternative hypothesis (i.e. significant correlation) than the null hypothesis (i.e. no correlation).

## 4. Discussion

As with humans, chimpanzees and cuttlefish, intelligence in Eurasian jays correlates with the capacity for self-control, particularly with the ability to wait for better possibilities. In the physical cognition experiment, performance varied across individuals. Individual performance was correlated across the five cognitive tasks, which suggests that there was a general intelligence factor underlying the jays' performance. In the self-control experiment, all the jays were able to delay gratification and wait for better possibilities when it led to a preferred food item. Nevertheless, the ability to exert self-control also varied across individuals. When subjects were offered a choice between their most preferred and second preferred food items, delay maintenance durations ranged from 20 s to 320 s (mean ± s.e. = 72 s ± 28.12) for the preferred but delayed option. When subjects were offered a choice between their most preferred and third preferred food items, delay maintenance durations ranged from 40 s to 160 s (mean ± s.e. = 76 s ± 11.30) for their preferred option. Individuals that were tested in both food quality treatments (i.e. most preferred versus second preferred; most preferred versus third preferred) exhibited longer waiting times when the immediate option was the third preferred food item, suggesting that the third preferred food item was less tempting and thus individuals could delay gratification for longer. Thus, we expected the wait times to be significantly shorter in the preferred versus second preferred treatment compared to the preferred versus third preferred treatment. While this was the case, however, the differences were not found to be statistically significant between the treatments. One explanation for the lack of statistical significance may be that the subject Jaylo, who exhibited the greatest self-control in the preferred versus second preferred treatment, did not complete all the trials in the preferred versus third treatment. We stopped testing Jaylo in this latter treatment because she stopped consuming the immediate reward in the control trials, suggesting she no longer valued the immediate food item. Inclusion of her results in one treatment and not the other probably influenced the difference between the strength of evidence observed between the two treatments (i.e. contrary to what we would expect the BF was higher in the preferred versus second preferred treatment). Furthermore, Jaylo's exclusion from the preferred versus third preferred treatment might have also contributed to the lack of statistically significant difference between the treatments. Nevertheless, performance in the physical cognition tasks was correlated significantly with the jays' ability to wait for better possibilities, whereby more intelligent jays showed greater self-control. These results highlight that a relationship between self-control and overall intelligence exists across disparate animal groups.

Advanced self-control in both humans and other animals is thought to be advantageous, particularly within specific social and ecological contexts. For example, within a social context greater self-control might have evolved in species that form strong social bonds. Individuals that temporarily deny their impulses to help social partners might strengthen social relationships as well as secure reciprocated favours in the future [44]. Within an ecological context, advanced self-control might have evolved in species that rely on variable food resources that are difficult to obtain or risky to retrieve owing to fierce resource competition or predation risk [30]. These pressures might have driven the evolution of extractive foraging (i.e. the capacity for tool use), caching behaviour, [30] or sit-and-wait foraging tactics to minimize detection from predators [19]. Such challenges are likely to have driven the evolution of advanced self-control in Eurasian jays since this ability has obvious benefits for their social and ecological behaviours including strengthening bonds with their partners, caching, and preventing others from stealing their caches [45]. One vital parameter determining the jays’ decisions to wait for better possibilities was the duration of the delay. Our results demonstrate that waiting appeared to be more difficult with increasing delay durations since the jays were more likely to succumb to consuming the immediate option as delays increased. Similar patterns have been observed in other large-brained bird species [26,27,29], primates [24,46–48] and cuttlefish [19].

Importantly, consistent exhibition of longer wait times may not always directly indicate greater cognitive complexity. For instance, in our study, subjects would often attempt to wait for preferred food items at roughly their maximum personal wait time and only abandon waiting if the delay continued beyond this. This suggests that the jays may have been unable to accurately anticipate the expected delay duration, otherwise we might expect them to abandon the task earlier. Indeed, waiting for better possibilities might not always be the optimal decision and thus flexible deployment of self-control is crucial. Under certain circumstances, favouring the immediate option can be beneficial, for instance, in times of high competition over resources [49,50] or if a predator is nearby [51]. However, the jays' exhibition of a fairly consistent maximum wait time may have been an artefact of our controlled laboratory conditions (i.e. a lack of competition and predation).

Nevertheless, the jays in our study were able to flexibly adjust their self-control behaviour in response to different circumstances. In the self-control experiment, the control condition offered a choice between an immediate less preferred option and a delayed preferred option that was visible but never obtainable. Here the jays always favoured the immediate option. By contrast, in the experimental condition, individuals were faced with a choice between two accessible options with differing delays. Under these circumstances, the jays were more likely to favour the more valuable outcome (i.e. preferred food) through tolerating a delay. These results demonstrate that Eurasian jays can flexibly trade-off between immediate and future options in relation to both the length of the delay and the final outcome. Furthermore, our results show that even among the more intelligent jays, performance in the experimental condition occasionally reflected failure to delay, which might reveal the intermittent need to disengage from self-control behaviour. The constant effort to wait for better possibilities can be stressful [52,53] and sometimes taking an immediate reward might increase future success in delaying gratification for better but delayed rewards [53]. This intelligent decision-making reflects the right balance between delaying gratification when it is best warranted but not when it is least warranted.

There is long-standing evidence of a relationship between intelligence and the ability to exert self-control in humans [9,54–56] and several cognitive abilities have been proposed to support this relationship [12]. For example, working memory, the amount of information that can be held in the mind during the execution of a cognitive task, has been linked to performance in delay of gratification tasks. Specifically, imposing working memory load can increase a subject's preference for smaller, immediate rewards over larger, delayed ones [57]. Not only do participants have to actively maintain representations of reward value in working memory but they also need to manage and integrate considerable amounts of complex information through concurrent cognitive processes [12]. Especially processes that are relevant to deciding between the options under consideration including strategy deployment (i.e. evaluating the opportunity cost of not taking the immediate option), affect management (i.e. controlling excitement over the prospect of obtaining the immediate option) and both the recollection of previous choices and anticipation of future choices. In a similar vein, self-control might require intellectual abilities that assist in the interplay between cognitive and affective types of executive control. For example, children with higher intelligence are more adept at shifting attention away from the affective properties of the rewards, which increases the tendency to choose delayed rewards [58]. Furthermore, individuals with higher intelligence tend to be better at transforming reward representations to make them more abstract, which leads to favouring better but delayed options [59]. Finally, individuals with higher intelligence are often more likely to adopt a premeditated, controlled manner of processing during certain cognitive tasks [60], with evidence suggesting that such an approach is linked to greater self-control. For instance, one study found that the ability to exert self-control increased when subjects were instructed to provide reasons for their choices [61].

Although it is less clear which cognitive abilities moderate the relationship between intelligence and self-control in non-human species, our results demonstrate that such an association exists in jays, a species far removed from the primate lineage. This suggests that the role of self-control is foundational in other cognitive abilities: specifically, individuals that were better at waiting for future rewards also performed better in the physical cognition tasks. One possibility for this significant correlation is that more intelligent jays learned the contingencies of the delay maintenance task more quickly and thus demonstrated greater optimal performance. However, all cognitive tasks in the physical cognition experiment were comparably related to performance in the self-control experiment and crucially four out of five of these tasks (i.e. spatial memory, object permanence, generalization learning and discrimination learning) did not directly assess any form of behavioural inhibition. This strongly suggests that self-control and intelligence have a common cognitive denominator in jays that is not solely linked by processes that involve the capacity for inhibition.

The small but growing body of evidence demonstrating that such a relationship is shared across a diverse range of taxa [18,19] suggests that strong selective pressures have shaped cognitive processes that rely on self-control or inhibition of action. Moreover, strong pressures might have positively selected for cognitive processes that act as precursor mechanisms to assist individuals with evaluating present and future options during complex decision-making. Given that corvids diverged from the primate lineage approximately 300 Ma, our findings suggest that advanced self-control and its relationship to other cognitive abilities evolved via convergent evolution. Evolutionary convergence occurs when distantly related species independently evolve similar traits to adapt to similar challenges. Similar cognitive traits arising in an even further removed species, the cuttlefish [19], which shared a common ancestor with vertebrates more than 550 Ma, provide additional evidence that such an association evolved multiple times, independently. This certainly makes the relationship between self-control and intelligence an interesting avenue for further research within comparative psychology.

In conclusion, we demonstrate how responses to delay of gratification tasks offer key insights into the nature of intelligence. Specifically, jays show a preference for delayed rewards as a function of condition, length of delay and intelligence. Investigating individual differences in self-control within and across species might help us gain a stronger understanding of other variations in broader aspects of cognition. Our results support previous findings showing that self-control is inextricably linked to other cognitive abilities in disparate animal groups. Worth noting is that this relationship has thus far been identified in species that possess advanced cognitive abilities. Therefore, this paradigm offers one potential avenue towards mapping how complex cognition evolved in the animal kingdom.

## Data Availability

The data are provided in the electronic supplementary material [62].
